# Radial Growth of Two Dominant Montane Conifer Tree Species in Response to Climate Change in North-Central China

**DOI:** 10.1371/journal.pone.0112537

**Published:** 2014-11-13

**Authors:** Yuan Jiang, Wentao Zhang, Mingchang Wang, Muyi Kang, Manyu Dong

**Affiliations:** 1 State Key Laboratory of Earth Surface Processes and Resource Ecology, Beijing Normal University, Beijing, China; 2 Key Laboratory of Traditional Chinese Medicine Protection and Utilization, Beijing Normal University, Beijing, China; 3 College of Resources Science and Technology, Beijing Normal University, Beijing, China; 4 Beijing Municipal Research Institute of Environmental Protection, Beijing, China; Chinese Academy of Sciences, China

## Abstract

North-Central China is a region in which the air temperature has clearly increased for several decades. *Picea meyeri* and *Larix principis-rupprechtii* are the most dominant co-occurring tree species within the cold coniferous forest belt ranging vertically from 1800 m to 2800 m a.s.l. in this region. Based on a tree-ring analysis of 292 increment cores sampled from 146 trees at different elevations, this study aimed to examine if the radial growth of the two species in response to climate is similar, whether the responses are consistent along altitudinal gradients and which species might be favored in the future driven by the changing climate. The results indicated the following: (1) The two species grew in different rhythms at low and high elevation respectively; (2) Both species displayed inconsistent relationships between radial growth and climate data along altitudinal gradients. The correlation between radial growth and the monthly mean temperature in the spring or summer changed from negative at low elevation into positive at high elevation, whereas those between the radial growth and the total monthly precipitation displayed a change from positive into negative along the elevation gradient. These indicate the different influences of the horizontal climate and vertical mountainous climate on the radial growth of the two species; (3) The species-dependent different response to climate in radial growth appeared mainly in autumn of the previous year. The radial growth of *L. principis-rupprechtii* displayed negative responses both to temperature and to precipitation in the previous September, October or November, which was not observed in the radial growth of *P. meyeri*. (4) The radial growth of both species will tend to be increased at high elevation and limited at low elevation, and *L. principis-rupprechtii* might be more favored in the future, if the temperature keeps rising.

## Introduction

The radial growth of trees is highly sensitive to changing climate factors. Therefore, tree-ring analysis has been applied to reconstruct past climate conditions and to detect important historical climatic events since the early twentieth century [1], [2], [3], [4], [5]. With increased interest in climate change and in its impacts on ecosystems, tree-ring analysis has received broader interest in recent years because it indicates not only the relationship between radial growth and climate conditions in the past but also a possible response in forest dynamics to future climate change [6], [7], [8], [9]. According to the report from the Intergovernmental Panel on Climate Change (IPCC), the temperature in the temperate region in Eurasia, which includes North-Central China, has increased more than the global average since 1970 [10]. Some studies based on the Normalized Difference Vegetation Index (NDVI) in the growing season and the length of climatic growing season have demonstrated a lengthening growing season in the temperate region of China, to which the extension of spring and summer has largely contributed [11], [12], [13]. As a driving force for vegetation dynamics, climate change can lead to gradual change in population or community processes and even alter community composition over large areas [14], [15]. If the climate change in the temperate region of China occurs as described above, forests in this region will experience higher temperature, especially in the spring and summer. Analyzing the relationship between radial growth and climate will improve our understanding of how tree growth could respond to changing climate in the past, present, and future.

*Picea meyeri* and *Larix principis-rupprechtii* are evergreen and deciduous conifer species, respectively. Both are indigenous species in China and have similar geographical distribution [16]. In terms of ecology, the former prefers to grow in shady and humid habitats, whereas the latter prefers light habitat and has a relatively stronger drought tolerance [17]. They are the most dominant tree species within the cold coniferous forest belt ranging vertically from 1800 m to 2800 m a.s.l. in North-Central China [18], [19], [20]. In the forest communities, they grow as the dominant species not only separately but also mixed together. Given their wide distribution and large biomass in the region, these two tree species play important roles in soil and water conservation and carbon storage for the regional forest ecosystems.

Dendrochronology has been used as a tool to explore the ecosystem response to climate variability in Northern China [21], [22], [23]. *P. meyeri* and *L. principis-rupprechtii* have been recognized as good materials for dendroecological studies because of their good cross-dating characteristics and high sensitivity to climate. The study of the radial growth of *P. meyeri* was first reported at the beginning of this century and pointed out that the radial growth of this species was positively correlated with precipitation in February and May of the current year but negatively correlated with mean monthly temperature in May of the current year [24]. These were then supported by another research [25]. A recent study showed, however, another kind of growth-climate relationship of *P. meyeri* at relative high elevation in the region, namely a negative correlation between growth and precipitation in June and a positive correlation with temperature in May [26]. As for the growth-climate relationship of *L. principis-rupprechtii*, the studies have also produced different results [27], [28], [29].

Despite the above mentioned dendrochronological researches, the earlier studies on these two species focused only on the relationship between tree-ring width and climate parameters for each single species, respectively. Those comparing the growth-climate relationship between the two co-occurring species have remained sparse until now. Even though the case studies have discussed the growth-climate relationship for each of the two species, the samples were collected from different sites, and the results appeared to be inconsistent. This present study uses the Luyashan Mountains as the study area. We collected tree-ring samples along an altitudinal gradient. We did not try to reconstruct the climate in past but instead examined trends in the radial growth of co-occurring *P. meyeri* and *L. principis-rupprechtii* trees. We asked if the radial growth of the two species in response to climate is similar to each other and whether the responses are consistent along altitudinal gradients. We sought to assess the potential change of the forest in North-Central China and to predict which species might be more favored in the future driven by the changing climate. These results may, in turn, better the understanding of climate-driven population processes that are related to current and future management decision and practices.

## Materials and Methods

### Sampling sites

**Figure 1:**
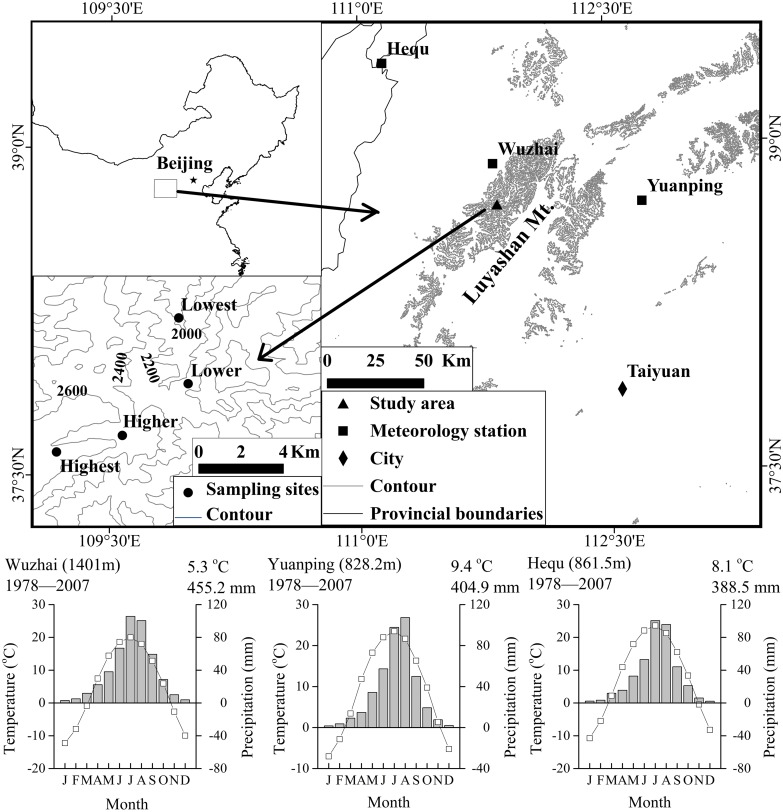
Location and climate survey of the study area.

The study sites were located in Luyashan Mountains, Shanxi Province (latitude between 38.67^°^ and 38.83^°^N and longitude between 111.83^°^ and 112.00^°^E), rising to an elevation of 2787 m at the summit. The climate type in this region is the warm temperate sub-humid climate according to Domrös and Peng [30]. The mean annual precipitation in the area is 455 mm with over 60% of this falling between June and September, and the mean annual temperature is 5.3°C, with a mean July temperature 20.0°C and mean January temperature −12.8°C (Fig. 1). The study area contains four vertical vegetation belts: the grassland and birch forest belt (1300–1500 m), the birch and poplar forest belt (1500–1900 m), the cold coniferous forest belt (1850–2700 m) and the sub-alpine meadow belt (>2700 m) at the peak area of the mountain [20]. The two dominant conifer tree species in the cold coniferous forest belt are *P. meyeri* and *L. principis-rupprechtii*. The soil under this vegetation belt is mountainous brown forest soil, a well-drained sandy loam characterized by 12–18% clay and 50–60% fine sand [31]. The pH of the topsoil was measured in August and September of 2009 and was between 6.4 and 7.3.

### Sample collection and process

Field work was conducted in the summer of 2009. No specific permissions are required for our conducting sample collection, since land in China belongs to the public and our field survey did not involve any endangered species. Based on the species altitudinal distribution, the tree-age classes, the dominant species composition, the site conditions and accessibility of the stands, four study sites were selected at different elevations. In detail, the lowest site was located at 1970 m, the lower site at 2240 m, the higher site at 2490 m, and the highest site at 2650 m. Each site was selected from the middle of the north-facing slope approximately 20–30^°^. According to Wilmking et al. [32], we avoided selecting sites at exposed locations, as they are not representative of the growth response of the whole landscape population. At each site, approximately 15–20 trees presumed with the eldest ages were selected as samples. Two increment cores were collected from each tree at breast height (1.3 m above the ground) (Table 1).

**Table 1:** Geographic location and forest stand characteristics of the sampling sites.

SiteCode	Longitude(E)	Latitude(N)	Elevation(m a.s.l.)	Slope	Density(Stems ha^−1^)	AverageDBH^a^ of*P. meyeri* (cm)	AverageDBH^a^ of*L. principis-rupprechtii* (cm)	AverageHeight^b^ of*P. meyeri* (m)	AverageHeight^b^ of*L. principis-rupprechtii* (m)
**Lowest**	38.78^°^	111.91^°^	1970	28^°^	450	10.7±0.9	8.9±1.0	5.2±0.4	5.0±0.4
**Lower**	38.75^°^	111.91^°^	2240	25^°^	317	18.4±0.8	24.6±3.1	12.5±0.5	17.8±2.8
**Higher**	38.73^°^	111.88^°^	2490	20^°^	608	15.3±0.5	20.6±2.3	10.4±0.2	14.1±1.6
**Highest**	38.72^°^	111.85^°^	2650	20^°^	608	17.3±0.7	15.9±1.0	10.0±0.3	8.8±0.7

a,b Data are presented as mean ± SE.

All tree-ring cores were visually cross-dated by the use of pointer years under a microscope. Ring widths were measured using the LINTAB measuring system with a resolution of 0.01 mm. The COFECHA program [33], [34] was used to test for avoiding possible dating or measuring errors. All cores with potential errors were rechecked and corrected if possible; otherwise, they were omitted from further analyses. Tree-ring width series were standardized to remove biological induced age-trends using the program ARSTAN [35], [36]. The detrended individual tree-ring series were then averaged to each species in each site by computing the biweight robust mean [37]. Considering the relatively young age of the trees and the important influence of precipitation on tree growth in the sub-humid region [38], we selected the residual chronology (RES) for the analysis, since the RES contains more high-frequency signals and is therefore better in examing the growth-climate relationship in the study area. The length of the eight chronologies varied from approximately 50 years to 80 years, but the common interval for all eight chronologies was determined to be 1978–2007 according to the age of the stand at the lowest site, the youngest of the four sites. The first 9 years of growth were excluded from the analysis because this period represented a time when the trees were very young and would have been more influenced by stand dynamics than climate.

### Meteorological data

The meteorological data for 1977–2007 were the averaged data from the three closest standard meteorological stations around the sampling sites, namely Wuzhai, Yuanping and Hequ stations (Fig. 1), to represent the complex montane climate in regional scale [39].

### Statistical analysis

Various statistical parameters were calculated to assess the reliability of the eight chronologies. Mean sensitivity (MS) was calculated for each series to indicate the relative changes in the ring width index variance between the consecutive years [40]. Inter-series correlation (R), the expressed population signal (EPS), and the signal-to-noise ratio (SNR) were calculated using a moving window approach. R is a measure of the common signal strength [41] that reflects the strength of impact of climate to some extent. EPS and SNR can reflect the signal strength of each chronology, considering the value of R and the number of samples [42].

Pearson’s correlation analyses between each pair of chronologies were performed to compare interspecific and intraspecific relationships of radial growth. Residual chronologies and climate relationships were also examined with correlations, which were calculated using tree-ring width indices and the monthly climate data, including monthly mean temperature and monthly total precipitation.

## Results

### Comparison of the chronologies between the two species

The common period for all chronologies was intended to be 1978–2007, based on the chronology of *P. meyeri* at the lowest site (P1), the shortest one among the eight. According to the results from ANOVA, the difference between the chronology parameters of the two species was not significant (Table 2). The chronologies demonstrated high-quality parameters, namely a high reliability with SNR values between 21.7–57.4 and EPS values (0.91–0.98) exceeding 0.85, which is the minimum threshold for a strong climate signal within the tree ring [42].

**Table 2:** Statistical parameters of each chronology.

Chronologies	Sample depth	Time period	MS	R	SNR	EPS
**P1**	40/20	1970–2007	0.25	0.51	10.5	0.91
**P2**	30/15	1917–2007	0.16	0.58	34.3	0.97
**P3**	42/21	1897–2007	0.17	0.45	26.4	0.96
**P4**	34/17	1947–2007	0.17	0.43	25.8	0.96
**L1**	42/21	1969–2007	0.16	0.25	7.3	0.88
**L2**	30/15	1924–2007	0.17	0.49	21.7	0.96
**L3**	42/21	1940–2007	0.15	0.66	57.4	0.98
**L4**	32/16	1947–2007	0.16	0.57	38.1	0.97
p-values resulted in one-way ANOVA on theparameters between two species			0.245	0.911	0.626	0.705

MS, mean sensitivity; R, inter-series correlation; SNR, signal to noise ratio; EPS, the expressed population signal; the R, SNR and EPS were all calculated for the common period, 1978–2007. P1, P2, P3, P4, the chronologies of *P. meyeri* in the lowest, the lower, the higher and the highest sites, respectively; L1, L2, L3, L4, the chronologies of *L. principis-rupprechtii* in the lowest, the lower, the higher and the highest sites, respectively.

To detect the similarity of the radial growth between *P. meyeri* and *L. principis-rupperchtii*, as well as that among the sites at different elevations, the Pearson’s correlation coefficients between each pair of chronologies were calculated (Table 3). The results indicated that trees of these two species grew in their particular rhythms in the lowest site at low elevation in comparison to those at other sites. The tree-ring indices of *L. principis-rupperchtii* at the lowest site positively correlated only to those of *P. meyeri* growing at the same site (*r* = 0.528, *p*<0.01). The indices of *P. meyeri* at the lowest site also correlated negatively to those of both species at higher elevation (*|r|*≥0.362, *p*<0.05). The chronology of *P. meyeri* at the lower site did not correlate to any of the other chronologies. The correlation between each pair of the chronologies at high elevation remained consistently positive to each other and became closer within species between different sites (*r≥*0.762, *p*<0.01) than between the species at the same site (*r≥*0.507, *p*<0.01).

**Table 3:** Pearson’s correlation coefficients between each pair of chronologies.

	L1	L2	L3	L4	P1	P2	P3	P4
**L1**		−0.290	−0.102	−0.109	0.528**	0.083	−0.214	−0.209
**L2**			0.686**	0.619**	−0.549**	0.100	0.256	0.303
**L3**		.		0.783**	−0.496**	0.124	0.507**	0.519**
**L4**					−0.495**	0.153	0.501**	0.538**
**P1**						0.003	−0.362*	−0.459*
**P2**							0.285	0.293
**P3**								0.762**
**P4**								

* and ** indicate the significant levels of 0.05 and 0.01, respectively; P1, P2, P3, P4, represent the chronologies of *P. meyeri* in the lowest, the lower, the higher and the highest sites, respectively; L1, L2, L3, L4 represent the chronologies of *L. principis-rupprechtii* in the lowest, the lower, the higher and the highest sites, respectively; the correlations are based on the common period 1978–2007.

### Similarity and difference of growth-climate relationship between the two species

**Figure 2:**
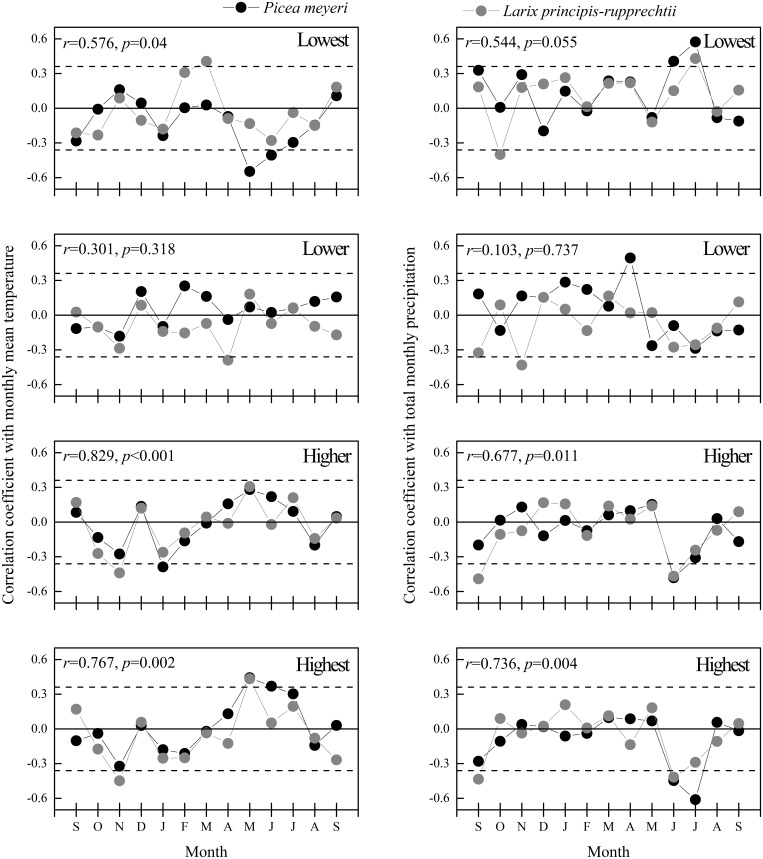
Results from correlation analyses on tree-ring width indices with monthly meteorological data. The dashed lines indicate a significance level of 0.05. *r* and *p* at the top left represent the correlation coefficients between the two data series in each field and the corresponding significance level. This level indicates the similarity of the growth-climate relationship between two species.

The growth-climate relationship was examined using the data of total monthly precipitation and monthly mean temperature for the period from 1978 to 2007. The similarity of the growth-climate relationship between the two species at each site was evaluated again through correlation analysis using two datasets, one of which was formed by the correlation coefficient between monthly meteorological data and tree-ring width indices of *P. meyeri* and the other by those of *L. principis-rupprechtii* (Fig. 2). The results indicated that *P. meyeri* and *L. principis-rupprechtii* displayed a similar response to the monthly mean temperature in radial growth at the lowest (*r* = 0.576, *p*<0.05), the higher and the highest sites (*r*≥0.767, *p*<0.01), whereas there was a response to total monthly precipitation only at the higher (*r* = 0.677, *p*<0.05) and the highest sites (*r* = 0.736, *p*<0.01). Both species appeared to have similar responses to total monthly precipitation at the lowest site, but the significance level for the test statistic reached, however, only to 0.1 (*r* = 0.544, *p*<0.1).

Although a similar relationship between meteorological data and radial growth was found at some sites, differences in the growth-climate relationship between the two species were also recorded in our study. In comparison to that of *P. meyeri*, the radial growth of *L. principis-rupprechtii* responded not only to meteorological data in the current growing season but also obviously to that of the previous autumn. The monthly mean temperature, for example, correlated negatively to the tree-ring indices of *L. principis-rupprechtii* in the previous November at the higher and the highest sites. In addition, a negative correlation between the total monthly precipitation and the tree-ring indices of this species appeared in autumn at all four sites (Fig. 2).

### Variation of radial growth in response to climate along elevation gradient

In addition to the similarity in the growth-climate relationship between the two species, the variations of radial growth in response to temperature and precipitation along the elevation gradient are also illustrated in the Fig. 2. In the current growing season, the radial growth of the two species in response to climate at high elevation appeared to be more or less similar, but this was not the case at low elevation. The radial growth of *L. principis-rupprechtii* responded to the monthly mean temperature in March positively at the lowest site, whereas *P. meyeri* responded in May negatively. The response to temperature became weaker in the medium elevation belt, represented by the lower site. At the highest site, the radial growth of both species correlated positively to the monthly mean temperature in May and *P. meyeri* and even to that in June as well. The variation of response to total monthly precipitation in the radial growth of the two species along the elevation gradient appeared to be more consistent than that to monthly mean temperature. The radial growth of the two species correlated to total monthly precipitation in July positively at the lowest site first and then became non-significant in the summer months at the lower site, and was further negative in June at the higher and the highest sites. In addition, a positive correlation between the total monthly precipitation and radial growth of *P. meyeri* in June and April occurred at the lowest and the lower sites, respectively, as well as a negative correlation in July at the highest site. As for the response to meteorological data in the previous autumn, none of the correlations between the meteorological data and the radial growth of *P. meyeri* were significant. The response to total monthly precipitation for the radial growth of *L. principis-rupprechtii* appeared to have a negative correlation in September, October or November and did not vary a great deal along the elevation gradient, whereas the correlation to monthly mean temperature in November was only noted at high elevation, namely, at the higher and the highest sites.

## Discussion

### Elevation-dependent growth variations and their relationship to horizontal zonal and vertical mountainous climate

The results of the Pearson’s correlation analysis showed various growth rhythms along the elevation gradient for both species (Table 3). These phenomena indicated an elevation-dependent divergence of radial growth for these two different coniferous species in the study area and demonstrated that the elevation of approximately 2200 m a.s.l., i.e., medium elevation, might be the vertical transition belt for the change of the growth trends. Another interesting feature in the variation of radial growth along elevation was that the two species grew in similar rhythms at sites the lowest, the higher and the highest sites but not the lower site. This finding again suggests, on one hand, the possible transition in radial-growth rhythms at the medium elevation belt, in which the lower site was located. On the other hand, this result revealed a phenomenon wherein different species could grow in synchronous radial-growth rhythms under constrained conditions, although they were different types of trees, i.e., evergreens and deciduous conifers.

As for the limiting factors constraining the growth at low and high elevations, it appears that these factors differed based on elevation. The lowest site was located in an area that was near the low limit of the vertical distribution for these species [20], [43]. The radial growth of both species there might be constrained by the drought resulting from the relatively higher temperature and lower precipitation, representing a more or less the horizontal zonal climate in the study area, which therefore resulted in special rhythm unique to this site. At high elevation, the constraining conditions on radial growth did not include drought but instead low temperature and high precipitation in the growing season (Fig. 2), which could lead to the shortage of thermal and radiation energy for photosynthesis, as well as a shorter growing season. The reasons for the change in limiting factors here could be attributed to the features of the mountainous climate characterized by increased precipitation and reduced temperature along the elevation gradient.

The radial growth of *P. meyeri* and *L. principis-rupprechtii* in the medium elevation belt, represented by the lower site, displayed interesting features. The two species had differing growth-climate relationships (Fig. 2). In addition, the growth of *P. meyeri* correlated to none of the patterns at the other sites at lower and higher elevations. These findings indicate that the two species in this altitudinal belt could grow with their own independent rhythms and might also indicate that there were optimal climate conditions at this site given that the climate factors did not limit the radial growth of the trees.

The above-mentioned elevation-dependent radial-growth rhythms suggest diversity in growth rhythms within a vertical cold evergreen coniferous forest vegetation belt. Since dendrochronological network could help to define bioclimatic zones and differential acclimation responses to spatial climate variation [44], [45], these multiform growth rhythms along the elevation might imply a typical transition of the growth-climate relationships in the case that a horizontal climate changed into a vertical montane climate.

### Radial growth in response to seasonal climate conditions

One of our findings in this study indicated that the growth-climate relationship of *P. meyeri* and that of *L. principis-rupprechtii* varied very similarly along the elevation gradient. The growth tended to correlate with temperature in some months from spring to summer negatively and precipitation positively in the lower altitudinal belt. At the high altitudinal sites, this situation was reversed. Another interesting finding was that the radial growth of *L. principis-rupprechtii* indicated negative responses both to temperature and to precipitation in the previous September, October or November, which was not observed in the radial growth of *P. meyeri*. Comparing these results with those from previous tree-ring studies on *L. principis-rupprechtii* conducted in Wutai and in Lüliang Mountains, located approximately 150 km away from our study area in the east and the south [28], [29], a significant correlation between radial growth and the climate factors in the previous autumn also could be detected. Even studies on another species within the larch genus, namely, *L. gmelini* in Northeastern China, have revealed a negative radial growth response to total monthly precipitation in the previous early autumn [46].

This phenomenon implies there are most likely different responses to climate in radial growth between the deciduous and evergreen coniferous tree species in North-Central China. The radial growth of deciduous coniferous trees, such as *L. principis-rupprechtii*, could be more dependent on the net primary production accumulated in previous growing season than in the current season during a certain period [47], [48]. In the current growing season, the deciduous trees can only provide with enough biomass through photosynthesis to support their radial growth just after they become needled. The energy and material required by the radial growth before, during or shortly after needle development might be mainly be sourced from the previous year’s production. Less precipitation in the early autumn before leaf falling might result in better solar and thermal conditions for photosynthesis [49], [50], which could, therefore, accumulate a larger amount of net primary production for insuring the growth in the next year [51].

### Possible growth trends driven by climate

Many previous studies have already revealed an air temperature increase in North-Central China and its nearby areas. Analysis of the long-term climate data indicates a lengthening of the growing season since the 1950s [13], [52], [53]. Several other studies using satellite-derived NDVI have identified an advancing in vegetation green-up since the 1980s in East Asia [11], [12], [54]. A phenological study in Beijing recently revealed further a significant advancement of the first bloom date, as driven by climate warming since 1990, by examining the First Flowering Data of 48 woody plants species [55].

**Figure 3:**
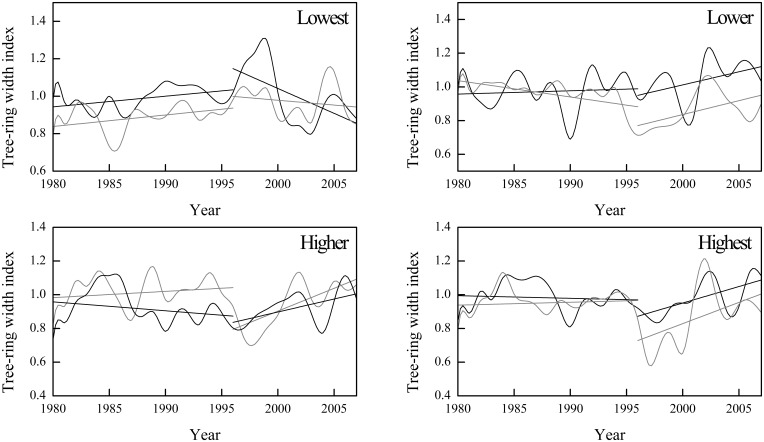
Trends in tree-ring indices of two species using a three-year average. The black lines represent trends of *P. meyeri*, whereas the grey lines represent those of *L. principis-rupprechtii*.

**Figure 4:**
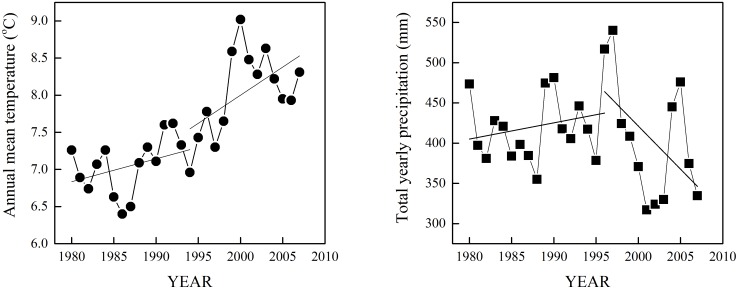
Trends of annual mean temperature and total yearly precipitation in the study area.

Considering this type of trend in temperature and its impact on plants, we analyzed trends in tree-ring indices in comparison with that in climate. The signatures of possible growth trends were consistent with changes in annual temperature and annual precipitation (Fig. 3). The climate data from our study area indicates a significant increasing trend in the annual mean temperature since the 1980s. The increase in temperature displayed an increasing tendency to become more apparent, and the total annual precipitation displays a falling trend from approximately 1995 (Fig. 4). Accordingly, a change in growth trends after 1995 could be observed at all four sites. At the lowest site, the growth trends of both species became a falling trend, and *L. principis-rupprechtii* had a smaller rate than *P. meyeri*. At other sites, both species displayed an increasing trend in radial growth, and the rate of *L. principis-rupprechtii* appeared to be greater than that of *P. meyeri* at the higher and the highest sites. These phenomena could imply that the radial growth of both species might increase at relatively high elevation and decrease at low elevation, and *L. principis-rupprechtii* might be more favored in the future, if the current climate trend continues.

## Conclusions

An elevation-dependent divergence of the growth rhythms for *P. meyeri* and *L. principis-rupprechtii* in the study area was clearly recognized in this study. The radial growth of *P. meyeri* displayed different annual patterns at lower, intermediate and higher altitudes. In addition, the radial growth of *L. principis-rupprechtii* indicated different patterns between the lower and the intermediate to higher elevations. The differentiations in radial growth were based on the different growth-climate relationship caused by the transition in climate types, namely, the transition from a horizontal climate to a vertical montane climate along the elevation gradient. The species-dependent different response to climate in radial growth appeared mainly in autumn of the previous year. The radial growth of *L. principis-rupprechtii* displayed negative responses both to temperature and to precipitation in the previous September, October or November, which was not recognized in the radial growth of *P. meyeri*. This phenomenon suggests that the climate conditions in the previous autumn played a more important role in the growth of deciduous trees than in that of evergreen trees. Furthermore, if the temperature in the North-Central China keeps rising whereas the precipitation continues to generally decrease, the radial growth of both species would tend to be increased at relatively high elevations and limited at low elevation. *L. principis-rupprechtii* might be more favored in the future, if the current climate trend continues. The large middle part of the vertical distribution belt (ranging from 2100 to 2500 m in elevation) for the species could provide the two species with the best climatic conditions and the most favorable habitats and thus offers both species the most appropriate sites for afforestation.

## Supporting Information

Dataset S1 **Tree-ring dataset.**
(TXT)
